# Supplementary material for: Pyruvate Dehydrogenase Kinase 1 inhibition mediated oxidative phosphorylation enhancement in cartilage promotes osteoarthritis progression
Source: BMC Musculoskelet Disord. 2023 Jul 20;24:597. doi: 10.1186/s12891-023-06585-6 (PMC10357736; doi:10.1186/s12891-023-06585-6)
Supplement: Supplementary file 1 — Additional file 1. [file 12891_2023_6585_MOESM1_ESM.zip › BP.Gsea.1653623667859/GOBP_TETRAPYRROLE_BIOSYNTHETIC_PROCESS.html]

Details for gene set GOBP\_TETRAPYRROLE\_BIOSYNTHETIC\_PROCESS[GSEA]

|  || Dataset | 55457\_collapsed\_to\_symbols.cls.cls #OA\_versus\_normal.cls.cls #OA\_versus\_normal\_repos |
| Phenotype | cls.cls#OA\_versus\_normal\_repos |
| Upregulated in class | OA |
| GeneSet | GOBP\_TETRAPYRROLE\_BIOSYNTHETIC\_PROCESS |
| Enrichment Score (ES) | 0.46704987 |
| Normalized Enrichment Score (NES) | 1.6461177 |
| Nominal p-value | 0.004608295 |
| FDR q-value | 0.21991454 |
| FWER p-Value | 1.0 |
Table: GSEA Results Summary

  

Fig 1: Enrichment plot: GOBP\_TETRAPYRROLE\_BIOSYNTHETIC\_PROCESS      
 Profile of the Running ES Score & Positions of GeneSet Members on the Rank Ordered List

  

| SYMBOL | TITLE | RANK IN GENE LIST | RANK METRIC SCORE | RUNNING ES | CORE ENRICHMENT || 1 | TMEM14A | transmembrane protein 14A [Source:HGNC Symbol;Acc:HGNC:21076] | 161 | 0.848 | 0.0835 | Yes |
| 2 | ALAS1 | 5'-aminolevulinate synthase 1 [Source:HGNC Symbol;Acc:HGNC:396] | 288 | 0.757 | 0.1595 | Yes |
| 3 | UROS | uroporphyrinogen III synthase [Source:HGNC Symbol;Acc:HGNC:12592] | 323 | 0.735 | 0.2401 | Yes |
| 4 | FECH | ferrochelatase [Source:HGNC Symbol;Acc:HGNC:3647] | 396 | 0.707 | 0.3146 | Yes |
| 5 | TSPO | translocator protein [Source:HGNC Symbol;Acc:HGNC:1158] | 692 | 0.595 | 0.3591 | Yes |
| 6 | TMEM14B | transmembrane protein 14B [Source:HGNC Symbol;Acc:HGNC:21384] | 841 | 0.557 | 0.4107 | Yes |
| 7 | UROD | uroporphyrinogen decarboxylase [Source:HGNC Symbol;Acc:HGNC:12591] | 905 | 0.541 | 0.4670 | Yes |
| 8 | ATP5IF1 | ATP synthase inhibitory factor subunit 1 [Source:HGNC Symbol;Acc:HGNC:871] | 1575 | 0.417 | 0.4624 | No |
| 9 | IREB2 | iron responsive element binding protein 2 [Source:HGNC Symbol;Acc:HGNC:6115] | 2215 | 0.337 | 0.4509 | No |
| 10 | ABCB6 | ATP binding cassette subfamily B member 6 (Langereis blood group) [Source:HGNC Symbol;Acc:HGNC:47] | 2717 | 0.278 | 0.4434 | No |
| 11 | CPOX | coproporphyrinogen oxidase [Source:HGNC Symbol;Acc:HGNC:2321] | 3110 | 0.239 | 0.4400 | No |
| 12 | SUCLA2 | succinate-CoA ligase ADP-forming subunit beta [Source:HGNC Symbol;Acc:HGNC:11448] | 3228 | 0.228 | 0.4567 | No |
| 13 | ALAD | aminolevulinate dehydratase [Source:HGNC Symbol;Acc:HGNC:395] | 3513 | 0.201 | 0.4574 | No |
| 14 | COX15 | cytochrome c oxidase assembly homolog COX15 [Source:HGNC Symbol;Acc:HGNC:2263] | 3727 | 0.183 | 0.4616 | No |
| 15 | PPOX | protoporphyrinogen oxidase [Source:HGNC Symbol;Acc:HGNC:9280] | 4225 | 0.135 | 0.4383 | No |
| 16 | PGRMC1 | progesterone receptor membrane component 1 [Source:HGNC Symbol;Acc:HGNC:16090] | 4636 | 0.101 | 0.4179 | No |
| 17 | ABCB7 | ATP binding cassette subfamily B member 7 [Source:HGNC Symbol;Acc:HGNC:48] | 4669 | 0.099 | 0.4266 | No |
| 18 | COX10 | cytochrome c oxidase assembly factor heme A:farnesyltransferase COX10 [Source:HGNC Symbol;Acc:HGNC:2260] | 6092 | -0.012 | 0.3174 | No |
| 19 | HMBS | hydroxymethylbilane synthase [Source:HGNC Symbol;Acc:HGNC:4982] | 6582 | -0.048 | 0.2849 | No |
| 20 | NFE2L1 | "nuclear factor, erythroid 2 like 1 [Source:HGNC Symbol;Acc:HGNC:7781]" | 6659 | -0.053 | 0.2850 | No |
| 21 | RSAD1 | radical S-adenosyl methionine domain containing 1 [Source:HGNC Symbol;Acc:HGNC:25634] | 7355 | -0.106 | 0.2431 | No |
| 22 | FXN | frataxin [Source:HGNC Symbol;Acc:HGNC:3951] | 8057 | -0.160 | 0.2067 | No |
| 23 | SPTA1 | "spectrin alpha, erythrocytic 1 [Source:HGNC Symbol;Acc:HGNC:11272]" | 8312 | -0.179 | 0.2072 | No |
| 24 | SRRD | SRR1 domain containing [Source:HGNC Symbol;Acc:HGNC:33910] | 8618 | -0.202 | 0.2065 | No |
| 25 | SLC6A9 | solute carrier family 6 member 9 [Source:HGNC Symbol;Acc:HGNC:11056] | 8704 | -0.209 | 0.2236 | No |
| 26 | SLC11A2 | solute carrier family 11 member 2 [Source:HGNC Symbol;Acc:HGNC:10908] | 9152 | -0.244 | 0.2165 | No |
| 27 | SLC25A38 | solute carrier family 25 member 38 [Source:HGNC Symbol;Acc:HGNC:26054] | 9904 | -0.312 | 0.1935 | No |
| 28 | ALAS2 | 5'-aminolevulinate synthase 2 [Source:HGNC Symbol;Acc:HGNC:397] | 10247 | -0.346 | 0.2061 | No |
Table: GSEA details [plain text format]

  

Fig 2: GOBP\_TETRAPYRROLE\_BIOSYNTHETIC\_PROCESS      
 Blue-Pink O' Gram in the Space of the Analyzed GeneSet

  

Fig 3: GOBP\_TETRAPYRROLE\_BIOSYNTHETIC\_PROCESS: Random ES distribution      
 Gene set null distribution of ES for **GOBP\_TETRAPYRROLE\_BIOSYNTHETIC\_PROCESS**
